# Modeling and Analysis of a High-Displacement Pneumatic Artificial Muscle With Integrated Sensing

**DOI:** 10.3389/frobt.2018.00136

**Published:** 2019-01-07

**Authors:** Hee Doo Yang, Brandyn T. Greczek, Alan T. Asbeck

**Affiliations:** Assistive Robotics Laboratory, Department of Mechanical Engineering, Virginia Tech, Blacksburg, VA, United States

**Keywords:** pneumatic muscle, textile actuator, soft actuator, robotic actuator, integrated sensing

## Abstract

We present a high-displacement pneumatic artificial muscle made of textiles or plastics that can include integrated electronics to sense its pressure and displacement. Compared to traditional pneumatic muscle actuators such as the McKibben actuator and other more recent soft actuators, the actuator described in this paper can produce a much higher (40~65%) contraction ratio. In this paper, we describe the design, fabrication, and evaluation of the actuator, as well as the manufacturing process used to create it. We demonstrate the actuator design with several examples that produce 120 and 300 N at pressures of 35 and 105 kPa, respectively, and have contraction ratios of 40–65%.

## Introduction

Robotic systems are becoming increasingly prevalent today, with possible applications including personal or mobile robots working alongside humans (Kim et al., 2013; Kristoffersson et al., 2013; Rus and Tolley, 2015) and exoskeletons worn to restore or improve human abilities (Dollar and Herr, 2008; Bogue, 2009; Lo and Xie, 2012; Shorter et al., 2013). While there are a variety of possible actuation schemes, pneumatic artificial muscles present some benefits as they can be very light-weight, compact, and use flexible materials. They can also be intrinsically compliant, providing natural human-robot interaction or increasing safety in the event of human-robot collisions. With these benefits, a number of groups have investigated different pneumatic actuator geometries and fabrication methods.

**Figure 1:**
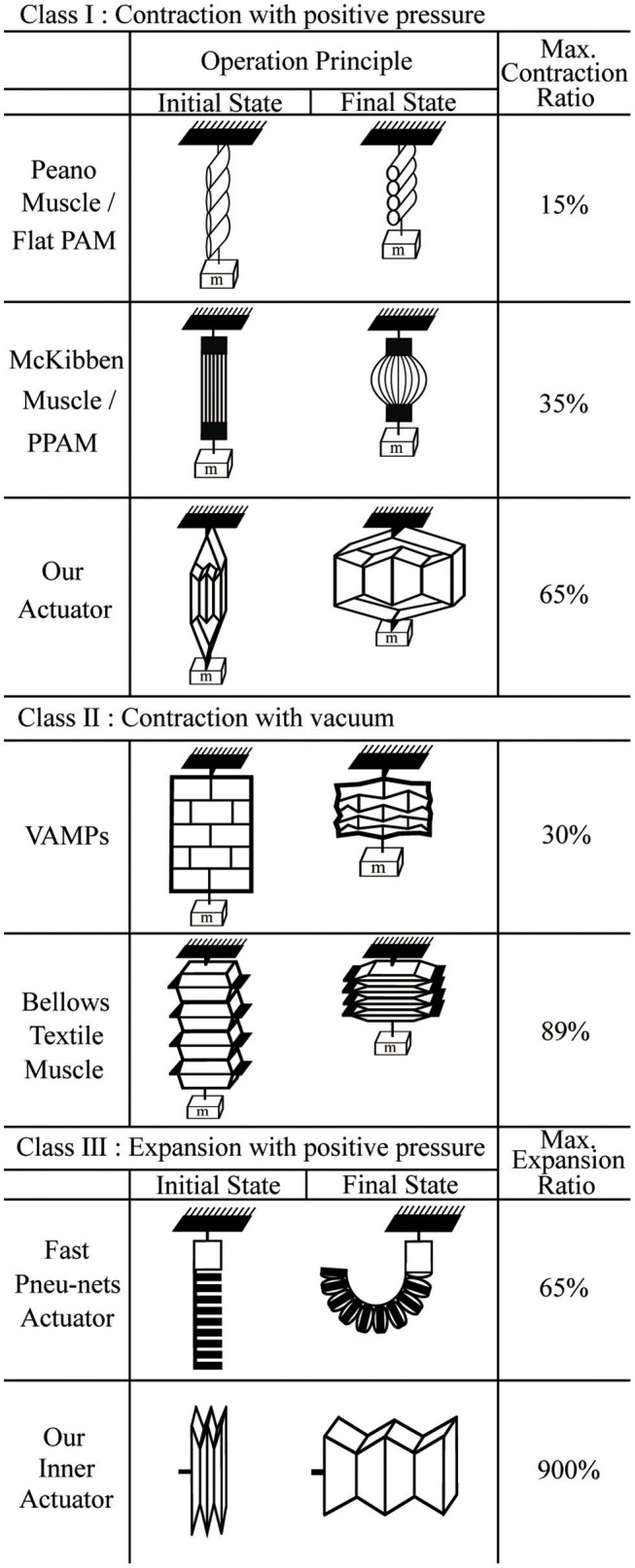
A classification framework for the different types of actuators. Class I actuators contract with positive pressure, Class II actuators contract with vacuum, and Class III actuators expand with positive pressure.

In the following paragraphs, we first review the state of the art in pneumatic actuators, and following this we describe our artificial muscle. We provide a classification framework for the different types of actuators in order to illuminate opportunities for new types of actuators. Figure 1 presents this classification framework and shows illustrations of the different types of pneumatic actuators described in the literature.

The first class of pneumatic actuators is those that contract when positive pressure is applied. The most well-known example of these is the McKibben actuator (Ching-Ping and Hannaford, 1996). The McKibben actuator has a structure consisting of an inner rubber tube with a mesh sleeve covering the tube. When the inner tube is pressurized, the mesh sleeve causes the structure to shorten; the specific shape is dictated by the mesh weave angle (Tondu, 2012). Many researchers have analyzed the McKibben actuator's properties, including the design, contraction velocity, contraction ratio, and operating force over a range of pressures (Daerden and Lefeber, 2000; Davis et al., 2003). For this paper, we define an actuator's contraction ratio as:

(1) $$\begin{array}{r} CR\left(\%\right)=\frac{\Delta k}{k_{0}}\times 100 \end{array}$$

where *k*_0_ is the initial length of the actuator and Δ*k* is the length it shortens during contraction.

However, the general McKibben actuator has some drawbacks: it has a low contraction ratio (30~35%) because of the geometry and the dead volume inside the actuator when uninflated, it exhibits friction between the mesh and inner tube that can lead to failures, and it requires deformation of the inner tube which can lead to fatigue and lower controllability at low pressures.

To solve the problems of friction and deformation of the inner tube, braided pneumatic artificial muscles (Tondu and Lopez, 2000; Davis et al., 2003; Davis and Caldwell, 2006; Doumit et al., 2009), and Pleated Pneumatic Artificial Muscles (PPAM) (Daerden and Lefeber, 2001; Daerden et al., 2001; Tondu, 2005; Vanderborght et al., 2008; Beyl et al., 2009, 2014; Villegas et al., 2012) have been developed. Their outer structures are different from the traditional McKibben muscle in that they are made of a single layer of aromatic polyamides, which eliminates friction between layers. These material properties give the PPAM increased performance and accuracy compared to the McKibben muscle, with a similar contraction ratio.

Another type of soft actuator is a Peano-fluidic muscle (Niiyama et al., 2014, 2015; Park et al., 2014; Veale et al., 2016) which is made of materials such as fabric or PVC film. Its structure consists of a few layers that lie flat in their initial state and are bonded at intervals in lines perpendicularly to the contraction direction. When air pressure is applied, the flat shape inflates to become round, causing the length of each of the tube segments to contract into its final state, giving a contraction ratio of 15–30%.

**Figure 2:**
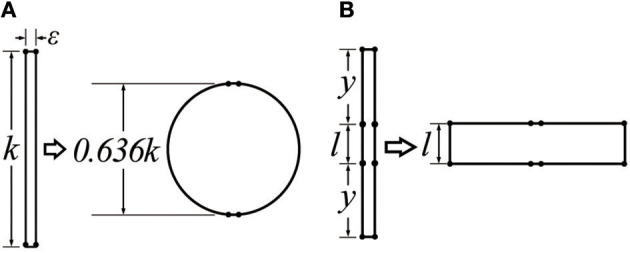
A schematic figure of the operating principle of mechanisms that contract when expanded horizontally, assuming a constant edge length. **(A)** Traditional pneumatic actuators start out tall and thin and contract to become more cylindrical or spherical; **(B)** greater changes in length can be achieved if the final state can be a wide rectangle instead of a circle. The pneumatic actuator developed in this paper uses this structure.

In each of these cases, the contraction ratio is relatively small because an initial shape that is relatively tall and thin is expanded to be more spherical or cylindrical; inextensible fibers convert this expansion in the lateral direction to a contraction in the lengthwise direction. This behavior combined with the fixed device circumference provides a fundamental limit on the contraction ratio. In the limit, in two dimensions a very tall and narrow rectangle with height *k*_0_ will expand at most to a circle with height (2/π)*k*_0_ = 0.636*k*_0_, corresponding to a contraction ratio of 36.4% (Figure 2A).

The second class of actuators in our framework includes those that contract when negative pressure (vacuum) is applied. In contrast to the actuators in the first class, there is no fundamental physical limit on the lengthwise contraction for a given actuator length. One example of this type is a bellows textile muscle (Belforte et al., 2014), similar to the Peano fluidic muscle. The actuator is made of fabric and constructed by connecting a number of round discs along their edges and centers. Since the actuator is made of textiles, it collapses to be extremely flat, giving it a contraction ratio of 89%. While not designed for this purpose, if used in expansion this would correspond to an expansion of up to 900% of its initial length.

Another example of this class is the Vacuum-Actuated Muscle-inspired Pneumatic structure (VAMP), which is a buckling pneumatic linear actuator (Yang et al., 2016). The actuator has a complex structure that includes vertical beams, horizontal beams, and collapsible air chambers. When vacuum is applied, collapsed air chambers cause the horizontal beams to buckle, resulting in a large length change in vertical direction. Since this actuator is made from silicone rubber, it cannot collapse as compactly as the bellows textile muscle, giving it a contraction ratio of 30%. Additionally, a different vacuum actuator called the Fluid-driven Origami-inspired Artificial Muscle (FOAM) was also recently introduced (Li et al., 2017). The actuator has the same principle as the VAMP, but it has a simple geometric design with a single skeleton structure inside of a pneumatic pouch, which allows the actuator to achieve high contraction ratios (90%).

While the actuators in this class can achieve impressive contraction ratios, they are fundamentally limited in the amount of force they can generate for a given actuator cross-section due to their use of negative pressure. Actuators powered by positive air pressure can potentially achieve extremely high pressures, but actuators depending on vacuum are limited to atmospheric pressure.

The third class of actuators is those that expand under positive pressure. An example of this type is the Fast Pneu-Nets Actuator (fPN) (Mosadegh et al., 2014; Wang et al., 2016), which is fabricated with a silicone elastomer. The actuator's structure is divided by two parts: a top layer that expands and an inextensible layer on the bottom. When positive air pressure is applied, flat chambers in top layer inflate to become round, pushing on adjacent chambers, and causing the structure to curl in conjunction with the inextensible layer.

A possible fourth class of actuators is those that would expand when provided with negative pressure (vacuum), although we did not find any examples of this in the literature, presumably because the benefits of such an actuator would not be significant. An example of a structure that would accomplish this behavior is a spherical structure that transforms into a cylinder when actuated, extending lengthwise; such a structure would likely need to be made from a polymer or have a relatively rigid internal structure to enforce the desired geometric transformation.

In summary, the pneumatic actuators in each of the four classes have different strengths and limitations. The actuators in Class I can have high force densities, but frequently suffer from relatively small contraction ratios. In Classes II, the force density is limited by atmospheric pressure, although large contraction ratios are possible. Actuators in Class III have usually been made to bend instead of extend linearly so the motion is constrained.

This paper presents a pneumatic actuator that overcomes many of the shortcomings of prior work. In particular, the actuator can achieve very high contraction ratios (40~60%) through a mechanism similar to that in Figure 2B. By creating an actuator structure that forms a wide rectangle instead of a sphere (in two dimensions) when expanded, much higher contraction ratios can be achieved. Additional advantages are that it collapses to be flat; it does not have friction between an outer actuator and inner actuator that may decrease its lifetime; it has a simple manufacturing process; and it can include integrated electronic sensors. The latter is possible because the actuator is constructed from a textile or plastic sheet and is designed so the material bends but does not stretch during operation. The remainder of this paper is organized as follows. We introduce the concept of the pneumatic actuator and its geometry in section Overview. We then discuss the fabrication process in section Fabrication Method, show a mathematical model of the actuator in section Actuator Modeling, and present results of experiments characterizing the performance of the actuator in section Experimental Evaluation. Finally, in section Electronics Integration and Evaluation, we present the fabrication process for modifying the actuator to include integrated electronics for controlling and monitoring displacement and pressure in real time.

## Overview

**Figure 3:**
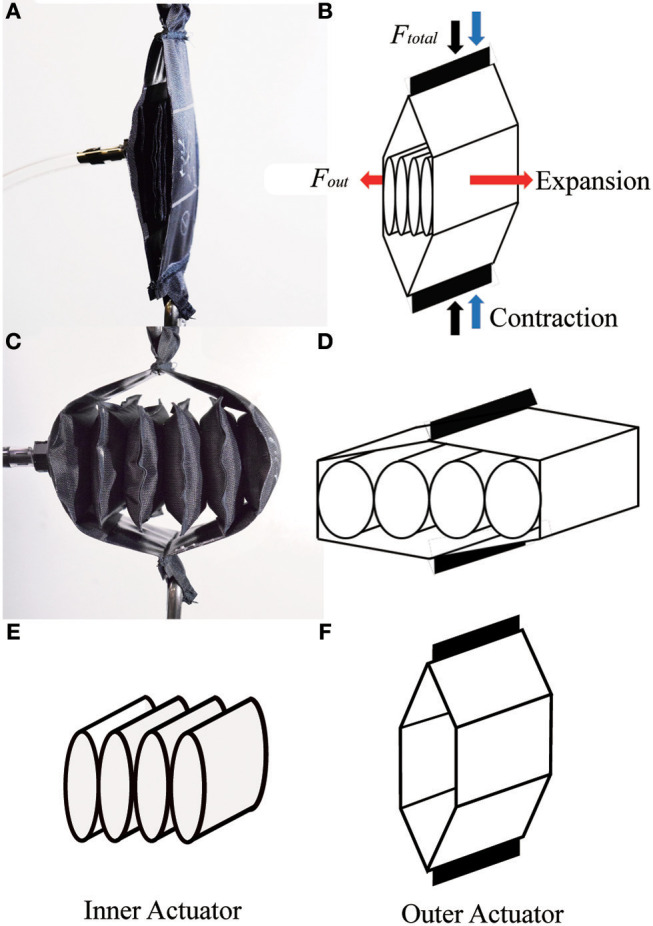
The concept of the actuator: **(A)** initial (uninflated) state of actuator prototype; **(B)** initial state of the actuator model; **(C)** final (inflated) state of actuator prototype; **(D)** final state of actuator model; **(E)** inner actuator; **(F)** outer actuator.

The pneumatic actuator described in this paper is shown in Figure 3, and is similar in concept to prior work by our lab (Yang, 2017) and others (Han et al., 2018). It consists of an “inner actuator”: formed by a series of connected chambers, and an “outer actuator” composed of two additional pieces of material (Figures 3E,F, respectively). In the initial state with no air pressure, the actuator is flat (Figure 3A), and the structure creates no force. When positive air pressure is applied to the inner actuator, the inner actuators inflate, and expand horizontally. The outer actuator then converts the horizontal motion of the inner actuators to a motion that contracts the actuator vertically (Figure 3C). The resulting structure is similar in geometry to a linkage used with piezoelectric energy harvesters to maximize their force (Conway et al., 2007; York et al., 2017). The overall scheme of the actuator is illustrated in Figure 3B. If a structure that is initially thin and flat expands into a rectangular structure, it can achieve a very high contraction ratio. Our actuator and the inner actuator as a standalone device are also shown in Figure 1, in Classes I and III, respectively.

The actuator can be made from thin plastic sheeting or from a textile coated with rubber or plastic so that it is airtight. A key feature of the actuator is that it does not require the material to stretch during its operation, a property it shares with other textile actuators such as the Peano fluidic muscle and the Bellow textile muscle. With this behavior, rigid or semi-rigid structures can be easily integrated with soft structures by simply affixing them to the material. Since the material does not stretch, the elastic modulus is similar to that of the rigid or semi-rigid materials, minimizing stress concentrations at the junction. We use this behavior to attach rigid electronic circuit boards to the actuator. In contrast, a structure made from a material like silicone that undergoes significant stretching will generate high stress concentrations at the interface with a rigid or semi-rigid material and may require intervening material with an intermediate stiffness (Mengüç et al., 2013).

**Figure 4:**
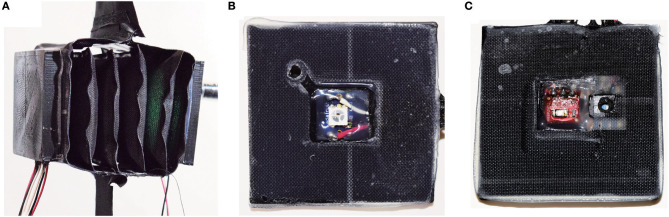
Example of the actuator including integrated electronics: **(A)** actuator prototype; **(B)** inside view of the plastic structure at one end with an embedded LED; **(C)** inside view of the plastic structure at the other end, including a phototransistor and a pressure sensor.

We present a version of the actuator that includes integrated electronics in Figure 4. The actuator has the same assembly process with the addition of two thin plastic structures, one at each end of the inner actuator. One plastic structure includes an LED board at the center, and the other includes a pressure sensor and an ambient light sensor to measure displacement in conjunction with the LED. These sensors provide information about the state of the actuator.

## Fabrication Method

**Figure 5:**
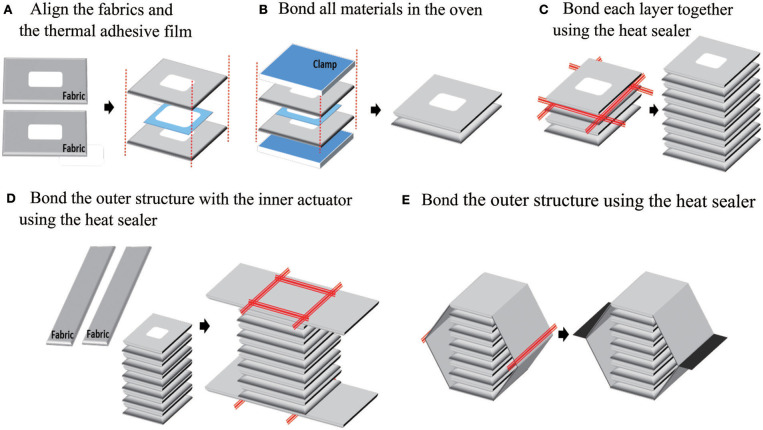
The manufacturing process of the actuator: to make the inner actuator, pieces of fabric and thermal adhesive film are aligned **(A)**, and then clamped and bonded in an oven **(B)**. Each chamber in the inner actuator is bonded at its edges with a heat sealer **(C)**. Next, the outer actuator is added by bonding two long strips to the ends of the inner actuator **(D)**, then bonding those together at their ends **(E)**, in both cases with a heat sealer.

We developed a simple fabrication process for the actuator (Figure 5) which was made of heat sealable oxford fabric with a urethane film coating on one side (Heat Sealable Oxford, 200 Denier, Seattle Fabrics, Inc.), along with a thermal adhesive film (Fastelfilm 20093, Fastel Adhesive, and Substrate Products, Inc.). The manufacturing process is separated into two parts: (1) bonding the inner edges of the actuator using a thermal adhesive film, and (2) bonding the outer edges of the actuator using a heat sealer. The fabrication process is divided into two parts because the fabric is only coated with the urethane film on one side; the uncoated side can only be bonded with the thermal adhesive film, while the coated side can only be bonded with the heat sealer.

In the first part, shown in Figure 5 steps A and B, the fabric sheets and thermal adhesive film layers are prepared according to the size of the inner connection parts and the number of inner layers. After the fabric and thermal film layers are cut, they are aligned, clamped together, and put in an oven at 250 for 10 min.

The second part of the fabrication process includes steps C–E in Figure 5. In step C, the outer edges of each pouch in the inner actuator are bonded using a heat sealer. In steps D and E, the outer actuator is bonded to the inner actuator and completed using the heat sealer.

## Actuator Modeling

**Figure 6:**
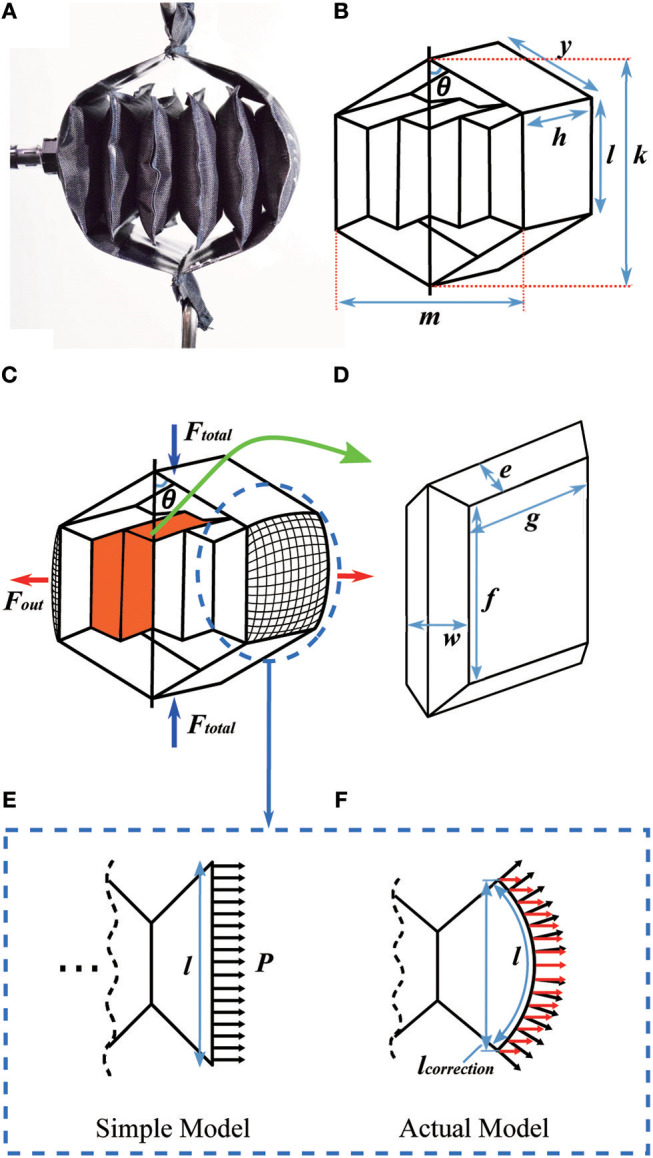
Detail on the mathematical model of the actuator. **(A)** Prototype of the actuator; **(B)** diagram of the actuator; **(C)** the inner structure of the actuator; **(D)** a single unit of the inner actuator; **(E)** the cross section of an end chamber using the simple model; and **(F)** the cross section of an end chamber using a model taking into account the actuator's curvature. In **(E,F)** black arrows show the pressure distribution over the actuator wall. In **(F)** the red arrows show the component of the pressure that contributes to Fout.

We next present a mathematical model of the actuator (Figure 6), which includes separate portions for the outer and inner actuators (Figures 6A,B, respectively).

### Outer Actuator Modeling

We first model the outer actuator, with the diagram and variables in Figure 6B. The inner actuator pushes on the middle portion of the outer actuator over a rectangular cross section, with a total outward force (*F*_*out*_), where

(2) $$F_{out}=Ph\;l$$

and *P* is the pressure inside the inner actuator, *h* is the width of the inner and outer actuators, and *l* is the height of the rectangle where the inner actuator connects to the outer actuator. If the actuator is made from a textile or other material that can bend, the simple actuator model in Figure 6B is not perfectly accurate at its ends. In this case, the region where the inner actuator connects to the outer actuator is not flat, but is a curved surface (Figures 6C,E,F), which results in an effective reduction in the inner actuator's height (*l*) and width (*h*) when the actuator is pressurized. Paulsen modeled the shape of a circular pouch (a Mylar balloon) when it is inflated, finding that the radius of an inflated pouch is about 0.7627 times the uninflated radius (Paulsen, 1994). While our pouches are rectangular instead of circular, the center of each side matches the model closely: we performed measurements of sample pouches and found they match Paulsen's model within 1%. Therefore, the true outward force of the inner actuator is based on a reduced cross-sectional area, where the effective height (*l*) and width (*h*) of the inner actuator are then *l*_*correction*_ = 0.7627*l*, and *h*_*correction*_ = 0.7627*h*. Thus, for inner actuators with flexible ends, the actual *F*_*out*_ highlighted in red (Figure 6F) is:

(3) $$F_{out}=Ph_{correction}l_{correction}$$

If the inner actuator is constrained to have rigid ends, for example by bonding pieces of plastic to them, then the simple model applies. The structure of the outer actuator (Figure 6C) leads to the following relationship between total contractile force from the outer actuator (*F*_*total*_) and the outward force from the inner actuator (*F*_*out*_):

(4) $$F_{total}=F_{out}\cot\theta$$

where θ is the angle between vertical and either of the top links in the outer actuator. We can calculate how much force the actuator will supply using (Equations 3 and 4) to obtain the following expression:

(5) $$F_{total}=Ph_{correction}l_{correction}\cot\theta$$

Equation (4) is used to choose the outer actuator geometry.

As shown in Figures 2B, 6B, the contraction ratio of the actuator is dictated by the ratio between the height of the inner actuator (*l*) and the length of the outer actuator between the top and the inner actuator (*y*). The initial actuator length is then *k*_0_ = 2*y*+*l*; when the actuator is fully contracted, the new length *k* = 2*y*cos*θ*+*l* is then approximately *l*, for a change in length of approximately Δ*k* = 2*y*. Similarly, the actuator is assumed to have an initial width of *m*≈0, and a maximum width of *m* = 2*y* when fully inflated. The contraction ratio can then be computed by plugging *k* and *k*_0_ into Equation (1). With reasonable choices for *y* and *l*, it is possible to achieve contraction ratios between 40 and 70%.

### Inner Actuator Modeling

**Figure 7:**
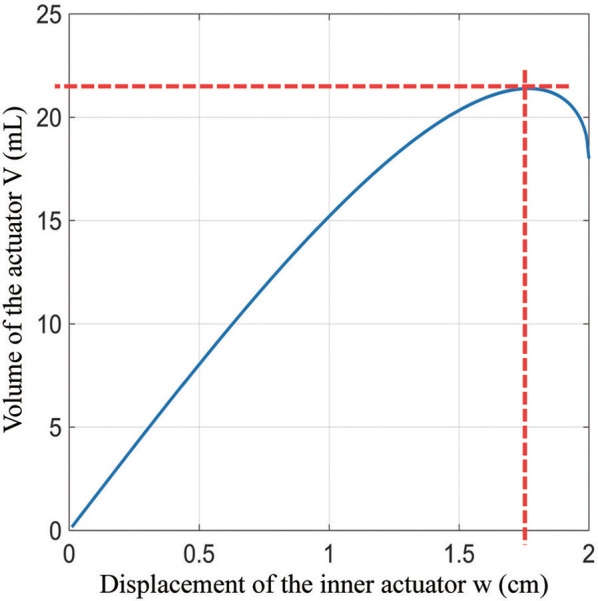
The relationship between width and volume of a single unit actuator.

Given the geometry of the outer actuator, we next describe how to design the inner actuator, including both the geometry and number of layers. We first model the inner actuator as a series of chambers connected together at their ends; Figure 6C shows one chamber in the inner actuator highlighted in orange, and Figure 6D shows a close view of the chamber. We model the chamber as a geometric solid with straight edges, although in reality the chamber will have complex three-dimensional curves. In this simplified model, the edges where the chamber is bonded to its neighboring chamber are of length *f* and width *g*, and the distance between the center connections and the edge is *e*, such that *l* = 2*e*+*f* based on the uninflated geometry. The distance across the chamber is denoted *w*, and it will vary as the chamber inflates. The actuator's maximum volume can be calculated using lengths *w*, *e*, and *f*. The volume of the single unit of the inner actuator is:

(6) $$V=\frac{4}{3}w\left(e^{2}-{\left(\frac{w}{2}\right)}^{2}\right)+wfg+w(f+g)\sqrt{e^{2}-{\left(\frac{w}{2}\right)}^{2}}$$

which is also shown in Figure 7 for sample lengths of *l* = *h* = 5 *cm, f* = *g* = 3 *cm*, and *e* = 1 *cm*. Comparing Equation (6) to Figure 6D, the first term is the volume of the corners, the middle term is the volume of the center portion, and the last term is the volume of the edges excluding the corners. The maximum volume inside the actuator as a function of the variables in Equation (6) cannot in general be found analytically, but it and the corresponding maximum width *w*_*max*_ = 1.77 *cm* (for this example) can be found numerically (Figure 7).

To validate the model, we experimentally measured several chambers from a sample inner actuator with those dimensions. Chambers in the middle of the actuator were found to have a thickness of 1.64 cm at 105 kpa, which is 8% smaller than the value predicted with the model. Pouches at the ends of the inner actuator were 2.13 cm thick, which is 20% larger than that predicted by the model. These differences are due to the fact that the pouches in the center of the actuator are constrained by their neighbors to be flat on both sides, while the pouches at the ends have one side that is unconstrained and can bulge outward. The errors from the pouches at the ends and middle tend to cancel, making the overall inner actuator length similar to that predicted by the model.

The maximum width gives the maximum amount each chamber in the inner actuator will expand during operation. To enable the outer actuator to have its maximum range of motion and expand to the width *m* = 2*y*, there must be at least N inner actuators, where for *y* = 3 *cm*,

(7) $$N=ceil\left(\frac{2y}{w_{max}}\right)=ceil\left(\frac{6}{1.77}\right)=ceil\left(3.38\right)=4$$

An actuator with this example geometry will have a peak actuation force *F*_*total*_ of 120 N at 34.5 kPa and 300 N at 105 kPa, and a contraction ratio of over 50%.

## Experiments

### Experimental Evaluation

We fabricated a variety of actuators with different geometries to characterize their performance and compare the effects of different parameters. For all of the actuators, we selected *h* = *l* and *f* = *g* to make the actuators have a square cross-section of the inner actuator, although the actuators do not need to have this in general. In most cases, *h* and *l* were chosen to be 5 cm to make the actuators relatively compact.

**Figure 8:**
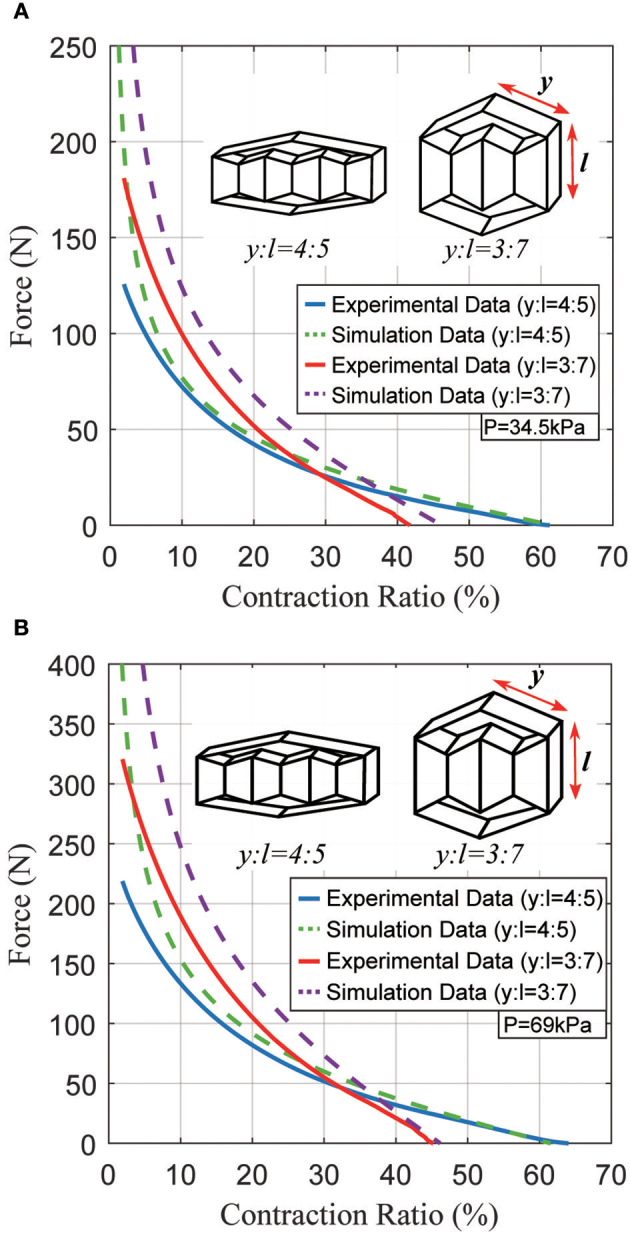
Typical static force created by the actuator as a function of the contraction ratio, for two different actuator geometries. **(A)** Results at a pressure of 34.5 kPa; **(B)** results at a pressure of 69 kPa.

In the first experiment, we fabricated two actuators with the same total length *k*_0_ but different ratios between *y* and *l*. We then used an Instron 4,204 machine to characterize the actuators' quasi-static forces and displacements. The actuators had dimensions of *y* = 4 *cm, l* = *h* = 5 *cm, f* = *g* = 3 *cm* for the first actuator; and *y* = 3 *cm, l* = *h* = 7 *cm, f* = *g* = 5 *cm* for the second actuator. Figures 8A,B show the force produced by each actuator at 34.5 and 69 kPa. The total length of each actuator is 13 cm, but there is a difference in contraction ratio due to the different ratios of *y*:*l*. The first and second actuators have contraction ratios of approximately 60 and 40% at 34.5 kPa, with forces up to 120 and 180 N, respectively. The difference in peak force is because the second actuator has a larger-area inner structure. With 69 kPa, two actuators generate approximately 1.8~2.0 times more force than those at 34.5 kPa with a slight increase in contraction ratios because the fabric does stretch slightly (1~2%) under high tensile forces.

**Figure 9:**
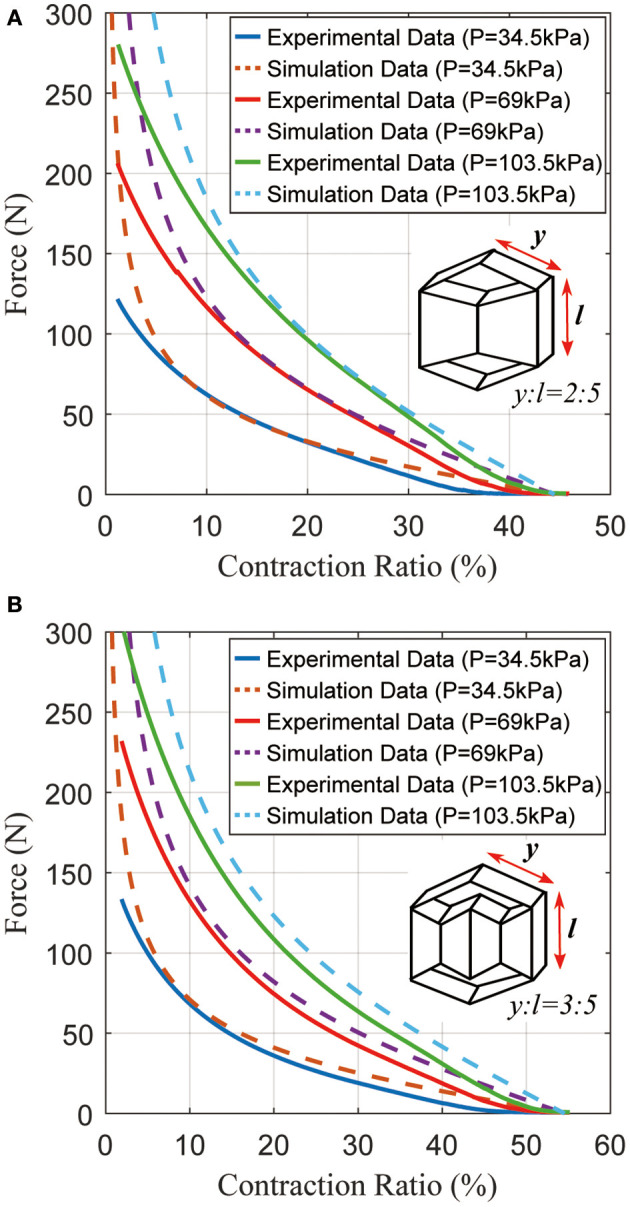
Typical static force created by the actuator as a function of the contraction ratio, with different pressures. **(A)** Results for an actuator with (*y*:*l* = 2:5); **(B)** results for an actuator with (*y*:*l* = 3:5).

A second experiment was performed to test two sample actuators under various pressures (34.5 ~ 103.5 kPa). In Figure 9A, an actuator with *y* = 2 *cm, l* = *h* = 5 *cm, f* = *g* = 3 *cm* was prepared. Depending on the pressure, the actuator can generate up to 125 N force at 34.5 kPa, and generate approximately 1.8 ~ 2.0 times more force whenever the pressure is doubled. As the pressure increases from 34.5 to 103.5 kPa, its contraction ratio only changes slightly, as expected. Figure 9B shows the results from a second actuator with *y* = 3 *cm, l* = *h* = 5 *cm, f* = *g* = 3 *cm*. According to the model, the actuator in Figure 9B should generate the same amount of force as the actuator in Figure 9A because their inner actuators are the same size. However, the peak force differs by 10~20 N due to imperfections in fabrication. Comparing Figures 9A,B, the peak force values are approximately equal, but there is a 10% difference in contraction ratios because of differing lengths (*y*). From the data, the pneumatic actuator can be competitive with the traditional McKibben actuator.

**Figure 10:**
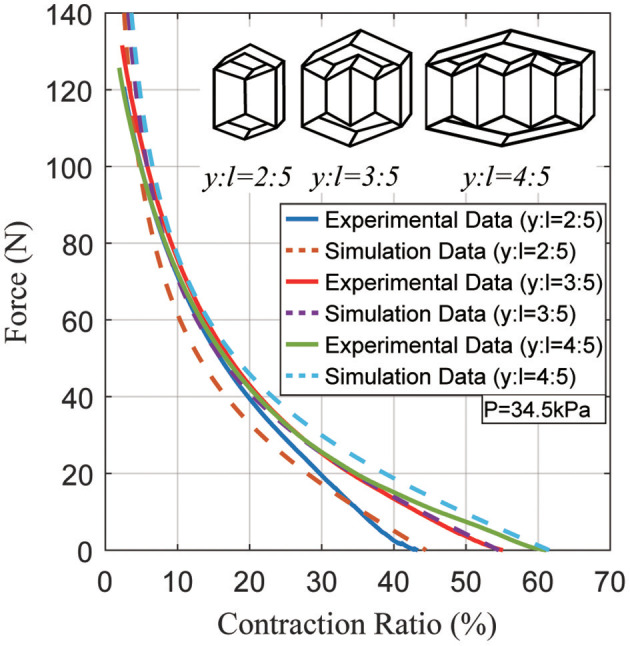
Typical static force created by the actuator as a function of the contraction ratio, showing the differences between several actuator geometries at a pressure of 34.5 kPa.

The third experiment compares three different actuator geometries at the same pressure. According to the actuator model, every actuator with the same inner actuator cross section generates the same peak total force (*F*_*total*_) with differing contraction ratios due to different lengths (*y*). Figure 10 shows the force vs. contraction ratio for several actuators with different lengths (*y*). The plot shows that indeed they can generate the same peak force, neglecting a 5~10 N error due to variations in fabrication.

### Electronics Integration and Evaluation

In order to use the actuator as a core component of a robot, the force and/or length of the actuator must be sensed. Instead of using external sensors, we accomplish this by embedding electronics within the actuator itself. Specifically, we sense the displacement by using an ambient light sensor (phototransistor) and light emitting diode (LED) at opposite ends of the inner actuator. This could also be accomplished by measuring the distance directly with an infrared or time-of-flight distance sensor; we use a phototransistor and LED because these may be able to be packed in a smaller area or volume than other sensors. In each of these, cases, optical distance sensors work well with this type of actuator because they can be completely enclosed by the actuator material, blocking ambient light. We also sense the pressure inside the inner actuator with a pressure sensor, which can be converted to the approximate force by knowing the outer actuator's geometry.

**Figure 11:**
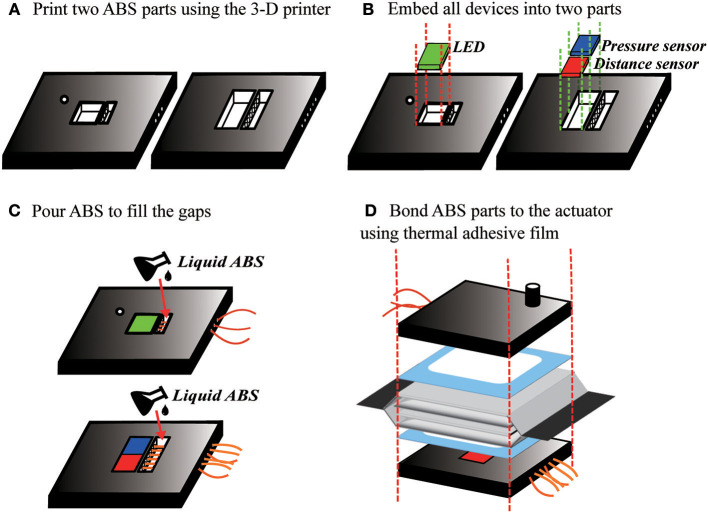
Fabrication process to include integrated electronics in the actuator. **(A)** ABS end pieces are made with a 3-D printer. **(B)** The electronics are placed into the end pieces, then **(C)** liquid ABS is poured in to fill the holes. **(D)** The end pieces are bonded to the actuator with thermal adhesive film.

To incorporate electronics, the fabrication process is modified slightly with additional steps to add the electronics as shown in Figure 11. First, we 3-D printed two acrylonitrile butadiene styrene (ABS) plastic parts to serve as substrates for the electronics on each end of the inner actuator. We embedded the LED in the center of one piece, and the ambient light sensor, and pressure sensor in the center of the other. In each side, the wires protrude through a hole in the back of the plastic part. To seal this hole and prevent air leaks, we poured liquid ABS, made by mixing Acetone and ABS material, into the hole around the wires, and waited for the liquid ABS to fully cure, around 24 h. Finally, we attached the plastic parts to the actuator using the thermal adhesive film.

We tested the sensorized actuator to determine the relationship between the distance of the outer actuator (*k*) and the displacement of the inner actuators (*m*), which can be used to precisely control the actuator. We determined this relationship using an Instron machine with an actuator geometry of *y* = 3 *cm, l* = *h* = 5 *cm, f* = *g* = 3 *cm*. During the tests we maintained a constant actuator pressure of 69 kPa while the Instron machine varied the actuatorḻength.

**Figure 12:**
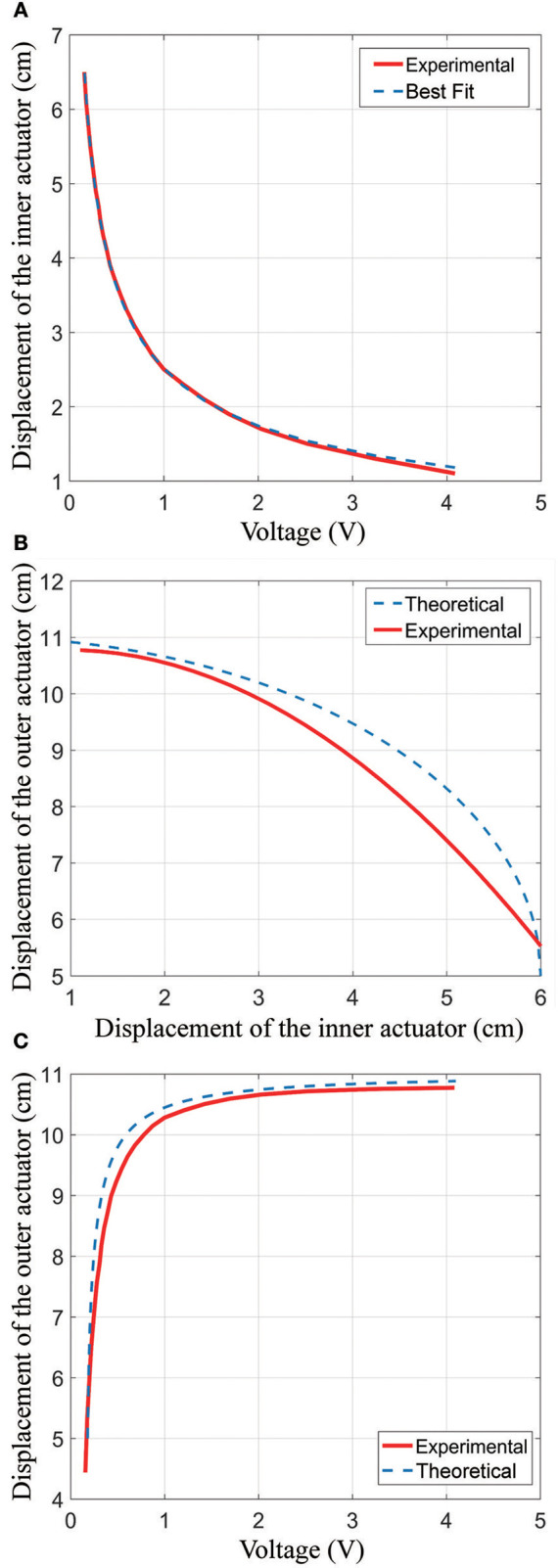
Results from an experiment showing how the true inner actuator length, true outer actuator length, and inner actuator length measurement are related. **(A)** The relationship between the inner actuator and voltage; **(B)** the relationship between the inner actuator and the outer actuator; **(C)** the relationship between the outer actuator and voltage.

Figure 12A shows the relationship between voltage (*V*) and the inner actuator length (*m*). To create this graph, the Instron machine was used to control the inner actuator length while the voltage output from the sensors was measured. Since the electronics are composed of an LED shining on a phototransistor, the amount of light falling on the phototransistor will vary as

(8) $$\text{m}=\text{a}\sqrt{\frac{1}{\text{V}}}+\text{b}$$

where *m* is the displacement of the inner actuator and *V* is the voltage output. This relationship exists since as the LED moves away from the phototransistor, the area on which the beam shines will increase with the square of the distance between them. Additionally, the voltage output of the phototransistor is proportional to the amount of light falling on it. Since it is very difficult to estimate the cross-sectional area of the phototransistor that will be sensitive to incoming light, we fit the measured curve to this form. The resulting best-fit line was:

(9) $$\text{m}=2.64\sqrt{\frac{1}{V}}-0.126$$

As shown in Figure 12A, this fit was very accurate, with a maximum error of 0.4 mm (0.8% of full scale) for displacements more than 1.7 cm and a maximum error of 0.8 mm (1.6% of full scale) for displacements of 1.2–1.7 cm.

Figure 12B shows the modeled (theoretical) and experimental relationship between the inner actuator distance and the outer actuator distance. For the Experimental line in the plot, we simultaneously measured the outer actuator length using the Instron machine and the voltage output from the integrated electronics, then computed the inner actuator distance using (Equation 9). The minimum length of the inner actuator is 1 cm because the fabric bellows structure has some thickness. In the plot, it can also be seen that the maximum inner actuator displacement is 6 cm, which follows from the geometry of the actuator.

To generate the “Theoretical” curve in Figure 12B, we used the geometry of the actuator in combination with equations in section Outer Actuator Modeling. The Theoretical curve thus has the equation:

(10) $$k=l+2\sqrt{y^{2}-\frac{m^{2}}{4}}$$

where *k* is the length of the outer actuator, *l* is the height of the rectangle, and *y* is the length of the outer actuator between the top and the inner actuator as shown in Figure 6B.

We plot the sensor output voltage (*V*) vs. the outer actuator length (*k*) in Figure 12C. These data were collected by moving the outer actuator with the Instron machine and measuring the resulting voltage from the electronics. The figure shows the experimentally-collected data as well as the “Theoretical” curve which was formed by combining the theoretical equation relating the outer actuator to the inner actuator (Equation 10) with the best-fit equation relating the inner actuator displacement and the measured voltage (Equation 9). The resulting equation is as follows:

(11) $$k=5+2\sqrt{3^{2}-1.74\left(\frac{1}{V}\right)+0.166\sqrt{\frac{1}{V}}}$$

where *k* is in cm. This relationship can be used to control the actuator.

Deviations between the theoretical model and measured relationship can be attributed to several causes. First, the theoretical model is based on a geometric shape with straight edges. In practice, the actuator chambers are curved, so the sides of the outer actuator are not perfectly vertical. This leads to a reduction in the outer actuator length as compared to what is predicted by the model, and indeed this can be observed in Figure 12B. Second, the dimensions of the fabricated actuator differed slightly from the intended lengths due to variations in manufacturing, leading to additional errors. In Figure 12B, we observe a maximum error of 8% comparing the theoretical equation to the experimental fit, which occurs at an inner actuator length of 5 cm and a voltage of 0.26 V. It is likely that the errors in this region are primarily due to the model (which assumed straight-line geometry) not matching the physical actuator, which has chambers that are curved to maximize the internal volume. In Figure 12C, we observe a maximum error of 5.2 mm (8.7% of full scale) comparing the voltage to the outer actuator length, at a voltage of 0.5 V. Notably, this model achieves a reasonable accuracy despite fitting only the voltage to inner actuator displacement, which can be easily measured in a lab environment. The necessity of fitting a curve to experimental data could be eliminated entirely by using an off-the-shelf distance sensor instead of a phototransistor-LED pair. Further improvements in modeling the relationship between the voltage and outer actuator displacement could be accomplished by fitting the experimental data directly.

**Figure 13:**
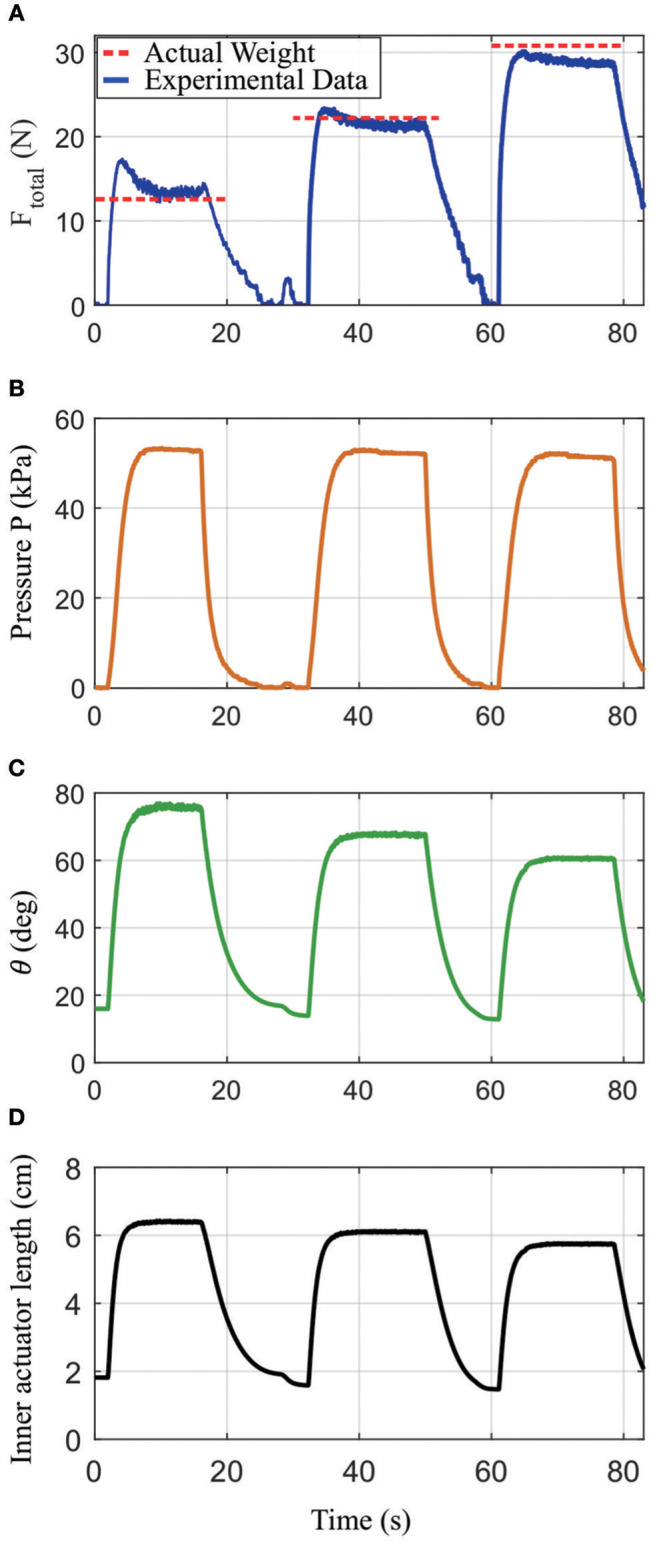
Results from an experiment showing how the actuator's output force can be calculated based on sensor readings from the distance sensor and pressure sensor combined with the actuator's geometry. **(A)** Calculated *F*_*total*_ under loads of 12.58, 22.21, and 30.79 N, respectively (blue solid line), as compared to the true applied forces (red dashed lines); **(B)** measured pressure in the inner actuator; **(C)** the angle between vertical and either of the top links in the outer actuator; **(D)** the measured inner actuator length.

Finally, the sensorized actuator was tested to monitor the approximate force by knowing the actuator's geometry and the internal pressure, with the results shown in Figure 13. These data were measured by inflating the actuator to around 52 kPa and lifting up 12.6, 22.2, and 30.8 N weights, respectively, then computing the force via *F*_*total*_ = *PA*cotθ. In this equation, **A** is the area of the inner actuator, which was measured experimentally by inflating the inner actuator to 50 kPa and having it push down onto a scale to measure the force, then using *A* = *F*_*out*_/*P*. In Figure 13A, steady-state forces of 12.6, 21.8, and 29.4 N were measured for the three loads, respectively, which corresponds to a maximum error of 4.6%. There are additional errors during inflation, which may be due to mismatches between the model and the actuator. The steady-state errors are due to imperfections in fabrication.

**Figure 14:**
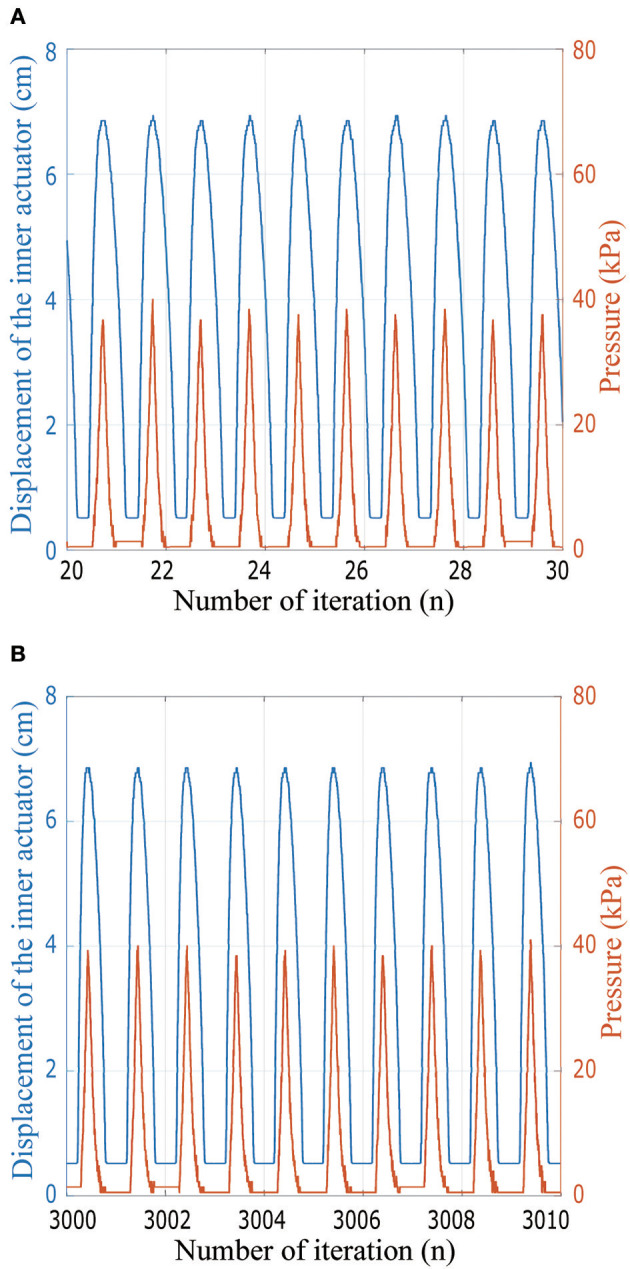
Experimental data from the cycling test of the actuator including the integrated electronics. The graph in **(A)** shows ten cycles soon after cyclic testing started, while in **(B)** the graph shows ten cycles after the actuator already completed 3000 cycles.

In addition, actuator cycling tests were performed with the results shown in Figure 14. While the actuator was tested for >3,000 cycles, a close-up view of several cycles is shown so the inner actuator length and pressure can be seen in detail. During the cycling test, the actuator shows consistent distance and pressure readings across multiple cycles. The pressure readings show fluctuations as the pressure drops off from the peak at each cycle; this is due to the tube connecting to the inflatable chamber being partially blocked by the actuator material as the air is drawn out of the chamber.

## Conclusions

In this paper, we present a high-displacement pneumatic artificial muscle actuator using soft materials that addresses some of the shortcomings of prior work. Compared to traditional pneumatic actuators, the actuator has a greater contraction ratio (40~65%), allowing for different geometries within a robot or exoskeleton. Compared to a similar actuator presented recently (Han et al., 2018), our actuator is fast to fabricate, can collapse to being flat, provides the opportunity for integrated sensors, and is much simpler to model. We present a model of the actuator so it can be designed for specified forces and displacements. Our tests showed that the actuator fits the model well, and the addition of electronics enables the force and displacement to be sensed. Future work includes making the electronics lower profile, understanding the different possible materials that can be used to construct the actuator, and improving the manufacturing process further.

## Author Contributions

HY: development of fundamental concepts; design, and construction of the actuators; acquisition and evaluation of experimental data; and written content. BG: construction of the instrumented actuator and the experimental setup, and testing. AA: development of fundamental concepts, project guidance, written content, and corrections.

### Conflict of Interest Statement

The authors declare that the research was conducted in the absence of any commercial or financial relationships that could be construed as a potential conflict of interest.
